# *Cryptococcus gattii* in Patients with Lymphoid Neoplasms: An Illustration of Evolutive Host–Fungus Interactions

**DOI:** 10.3390/jof7030212

**Published:** 2021-03-16

**Authors:** Olivier Paccoud, Marie-Elisabeth Bougnoux, Marie Desnos-Ollivier, Bruno Varet, Olivier Lortholary, Fanny Lanternier

**Affiliations:** 1 University of Paris, Necker-Pasteur Center for Infectious Diseases and Tropical Medicine, Necker-Enfants Malades University Hospital, Assistance Publique des Hôpitaux de Paris (AP-HP), 75015 Paris, France; olivier.paccoud@aphp.fr (O.P.); olivier.lortholary@aphp.fr (O.L.); 2 University of Paris, Department of Mycology, Necker-Enfants Malades University Hospital, Assistance Publique des Hôpitaux de Paris (AP-HP), 75015 Paris, France; marie-elisabeth.bougnoux@aphp.fr; 3 Molecular Mycology Unit, Centre National de la Recherche Scientifique (CNRS), National Reference Center for Invasive Mycoses & Antifungals (NRCMA), Pasteur Institute, UMR2000, 75015 Paris, France; marie.desnos-ollivier@pasteur.fr; 4 University of Paris, Department of Hematology, Necker-Enfants Malades University Hospital, Assistance Publique des Hôpitaux de Paris (AP-HP), 75015 Paris, France; bvaret@aphp.fr

**Keywords:** *Cryptococcus gattii*, cryptococcosis, haematological malignancy, lymphoid neoplasms, chronic lymphocytic leukemia, ibrutinib

## Abstract

Recent outbreaks of *Cryptococcus gattii* (CG) infections in North America have sparked renewed interest in the pathogenic potential of CG, and have underscored notable differences with *Cryptococcus neoformans* in terms of geographic distribution, pathogen virulence, and host susceptibility. While cases of CG are increasingly reported in patients with a wide variety of underlying conditions, only very few have been reported in patients with lymphoid neoplasms. Herein, we report a case of autochthonous CG meningitis in a patient receiving ibrutinib for chronic lymphocytic leukemia in France, and review available data on the clinical epidemiology of CG infections in patients with lymphoid neoplasms. We also summarise recent data on the host responses to CG infection, as well as the potential management pitfalls associated with its treatment in the haematological setting. The clinical epidemiology, clinical presentation, and course of disease during infections caused by CG involve complex interactions between environmental exposure to CG, infecting genotype, pathogen virulence factors, host susceptibility, and host immune responses. Future treatment guidelines should address the challenges associated with the management of antifungal treatments in the onco-haematological setting and the potential drug-drug interactions.

## 1. Introduction

Cryptococcosis is one of the most frequent causes of invasive fungal infections causing human disease, and the most common cause of meningitis in adults living with HIV in Sub-Saharan Africa [1,2,3]. Cryptococcosis is caused by *Cryptococcus neoformans* (CN) and *Cryptococcus gattii* (CG). While CN has by far been the focus of most research due to its importance as an AIDS-defining pathogen, the clinical epidemiology of cryptococcosis caused by CG is relatively less defined [4]. Recent outbreaks of CG infections in North America, primarily caused by a virulent strain of the VGII lineage, have sparked renewed interest in the pathogenic potential of CG and have underscored notable differences with CN in terms of geographic distribution, pathogen virulence, and host susceptibility [5]. Cases of both CN and CG have been reported in patients with haematological malignancy [6,7]. In particular, there have been sporadic reports of cases of cryptococcosis in patients receiving ibrutinib for lymphoid neoplasms. Herein, we report a case of meningitis caused by CG in a patient receiving ibrutinib for relapsed chronic lymphocytic leukaemia, and review the clinical epidemiology of CG infections during haematological malignancy. We also summarise recent data on the host responses to infections caused by CG and its management in the setting of haematological malignancy.

## 2. Case Description

An 88-year-old woman was referred to Necker-Enfants Malades Hospital in Paris, with a 5-day history of fever and headache. The patient had a history of relapsed chronic lymphocytic leukaemia (CLL), for which she had received successive treatment lines of chloraminophene, rituximab, chloraminophene, and lastly, rituximab and bendamustine. Due to relapse, the patient was currently receiving ibrutinib (420 mg/day), which had been started 8 months prior to admission. The patient resided in a house located in the south-east of France. She had no history of travel but reported extensive exposure to eucalyptus trees around and inside her house. At admission, we noted psychomotor slowing and meningeal syndrome. Blood counts showed neutropenia (0.5 × 10^9^ cells/L, reference range 4–10 × 10^9^ cells/L) and lymphopenia (1.4 × 10^9^ cells/L, reference range 1.5–4 × 10^9^ cells/L). C-reactive protein level was at 68 mg/L (reference < 5 mg/L) and CD4+ lymphocyte counts were at 186/µL (reference > 700/µL). Serum gamma globulins were markedly reduced with IgG levels at 0.99 g/L (reference range 6.6–12.8 g/L). Analysis of cerebrospinal fluid (CSF) showed pleocytosis with 116 cells/mL (96% lymphocytes), elevated protein levels (0.7 g/L, reference < 0.4 g/L), and hypoglycorrhachia (0.33 g/L, reference > 0.5 g/L). Theopening pressure of CSF was elevated (25 cm H_2_O, reference < 20 cm H_2_O). An India ink test on CSF was positive. *Cryptococcus* antigen (CALAS, Meridian Bioscience) was positive in the CSF (titre of 11) and in the serum (titre of 19). Fungal cultures of the CSF were positive and the MALDI-TOF method (Bruker Biotyper) allowed identification of the species *Cryptococcus gattii*. Genotyping was performed by multilocus sequence typing (MLST) at the French National Reference Center for Invasive Mycoses and Antifungals (NRCMA), using the ISHAM-7 loci scheme and comparisons with the online database (http://mlst.mycologylab.org/page/CG%20, main accessed on 22 October 2020) [8], which led to the identification of *Cryptococcus gattii* serotype B, belonging to the molecular clade VGI, genotype ST197. This genotype belongs to the European–Mediterranean cluster identified by Hagen et al. [9]. Mycological blood cultures were negative, and cerebral magnetic resonance imaging was unremarkable. Chest computed tomography images showed a 2 cm × 3 cm mass lesion of the upper left lobe, evocative of a CG cryptococcoma. Testing for anti-granulocyte-macrophage colony stimulating factor (GM-CSF) autoantibodies was negative. We administered intravenous liposomal amphotericin B (L-AMB) (3 mg/kg/day) and 5-fluorocytosine (5-FC) (100 mg/kg/day), and ibrutinib was suspended. After 14 days of combination induction therapy, neutropenia worsened (0.2 × 109 cells/L) and antifungal treatment was switched to oral fluconazole (800 mg/day). Four weeks after treatment initiation, neutrophil counts were within normal ranges (2.2 × 109 cells/L). At week 12, ibrutinib was reintroduced due to lymph node growth, albeit at a reduced dosage (140 mg/day) because of concerns regarding drug-drug interactions between fluconazole and ibrutinib. Repeated cryptococcal antigen assays at 3 months of treatment initiation showed marked reduction in titres (4 and 3.1 in the CSF and serum, respectively). The patient is currently still receiving ibrutinib, and has experienced no relapse of infection or CLL after more than two years of follow-up.

## 3. Clinical Epidemiology of *Cryptococcus gattii*

CG is an encapsulated basidiomycetous yeast from the *Cryptococcus* species complex, which grows preferentially in over 50 species of trees, including eucalyptus trees, and in their hollows [10,11]. The CG species comprises two serotypes, B and C, and can be further subdivided into five molecular lineages (VGI–V) [12], with VGV having only recently been identified in the Central Miombo Woodlands of Zambia, Africa [13]. Identification of CG at a molecular subtype level appears to be important, as these lineages seem to differ in geographic range, host susceptibility, and antifungal susceptibility [5,14,15]. Cases of endemic or sporadic CG infections have been reported for all molecular lineages, in a wide range of regions, including Australia and Papua New Guinea (VGI and VGII), South America—including French Guiana—(VGI–IV), Southeast Asia (VGI–IV), Southern and Central Africa (VGIV and VGV), and the United States (VGI and VGIII), as well as more recently in an outbreak setting in North America (VGIIa and VGIIb) [10,13,16,17,18]. In Europe, there have also been sporadic reports of CG, a majority of which are believed to be imported or travel-related, although autochthonous cases have been reported as well [9]. Indeed, there is increasing evidence that CG has been emerging in Europe over the past two decades [9,19], and a Mediterranean cluster belonging to genotype VGI was found in environmental and clinical isolates recovered from southern Italy, Portugal, and France [9,20].

Precise data on the incidence and clinical presentation of CG are biased by the absence of routine identification at a species level in most cases of cryptococcosis outside of endemic and outbreak settings [5,14]. Nevertheless, prior to the description of outbreak cases in North America in the early 2000s, the clinical epidemiology of CG was thought to be distinct from that of CN. Historically, infections by CG were believed to be geographically limited to tropical or sub-tropical regions [16]. In addition, whereas only 10–20% of patients with CN infections were seemingly immunocompetent, this was the case for up to 80–100% of patients with endemic CG infections [21,22]. Finally, CG more frequently caused focal pulmonary disease, cerebral cryptococcomas, hydrocephalus, and was more often responsible for neurological sequelae [21,22,23]. This clinical paradigm has since shifted with studies of outbreak cases that started in 1999 on Vancouver Island, British Columbia, Canada, and subsequently spread to the U.S. Pacific Northwest [24,25]. In these outbreak cases, 97% of which were caused by yeasts of the VGII lineage (primarily by VGIIa, and to a lesser extent by VGIIb), 38–59% of affected patients had an underlying condition (most frequently an underlying pulmonary disease or a history of systemic corticosteroid use) [26,27]. Compared to CG cases reported in other locations, as well as sporadic strains from North America (i.e., of molecular type VGI), VGII outbreak strains more often presented with pulmonary involvements and were associated with unusually high case fatality rates (8–33%) [26,28,29]. Nevertheless, although the infecting genotype seems to contribute to the phenotype of the infection, it now seems increasingly likely that clinical presentation is dictated by a more complex combination of factors that also includes host immune status and environmental exposure to CG [22,30].

## 4. Host Defences against *Cryptococcus gattii*

Of the more than 30 species in the *Cryptococcus* genus, only CN and CG are known to be pathogenic to humans [1]. While cryptococcosis caused by CN generally results from the reactivation of latent infection, almost exclusively in the setting of immunosuppression, CG is considered a primary pathogen [5]. Infection occurs via inhalation of yeast cells or basidiospores from the environment, with supposed subsequent survival in the lungs within intra-alveolar macrophages. Dissemination to the central nervous system can ensue, either via transcellular migration across the blood–brain barrier, or alternatively via a paracellular passage using a ‘Trojan Horse’ method, during which monocytes are hijacked by *Cryptococcus* spp. [31].

While the specific interactions between CG and the host immune system have not been studied to the same extent as for CN, many of these seem to be similar between the two species. They involve specific virulence factors, pathogen escape mechanisms, and alterations to innate and adaptive immunity [32,33]. The most studied of these virulence factors is the presence of a polysaccharide capsule, which provides evasion to immune recognition, decreases complement activity, and downregulates the host production of TNF-alpha and IFN-gamma [32]. Survival of the yeast during phagocytosis is further facilitated by a mechanism of capsule enlargement, as well as the ability to escape phagocyting macrophages through exocytosis [34,35]. Additional virulence factors include the antioxidant properties of melanin, which is present in the cell wall and protects the yeast from free radicals, and the ability to survive at body temperature [33]. In mice models of CN infection, depletion of specific cytokines has shown that lack of IL-12 and IFN-gamma production was associated with increased mortality, whereas downregulation of IL-13 and IL-4 promoted survival [35]. The fact that cases of cryptococcosis have been reported in patients with circulating antibodies against IFN-gamma and in those with IL12Rβ1-deficiency seems to support the crucial role of the IL-12/IFN-gamma axis in anti-cryptococcal immunity [36,37]. TNF-alpha appears to play an equally pivotal role in host responses to CN, illustrated by the observation that TNF-alpha-deficient mice displayed reduced survival to disseminated CN infections despite increased IL-12 and IFN-gamma levels [38]. Interestingly, the cytokine pattern involved in the host response to CG was shown to differ from that of CN. In vitro studies using heat-killed isolates incubated with human peripheral blood mononuclear cells have shown that CG infection, compared to CN infection, was associated with higher concentrations of the pro-inflammatory cytokines IL-1β, TNFα, and IL-6 [39]. This could help explain the severity of CNS disease oftentimes reported during non-outbreak CG infections in immunocompetent patients [40], which is characterised by high rates of hydrocephalus and immune reconstitution inflammatory syndrome (IRIS)-like manifestations [6]. It has also led to the suggestion that a substantial subset of apparently immunocompetent patients with CG infections could in fact have subtle immune defects. Evidence supporting this hypothesis can be found in reports of CG infections revealing subclinical antibody deficiencies [41], as well as the discovery of circulating auto-antibodies against GM-CSF in otherwise immunocompetent patients with CG meningitis [42].

Finally, as previously mentioned, one of the major differences between CG and CN appears to be the extent to which pathogenicity and clinical presentation of CG seem to be linked to infecting genotype. Reports of non-outbreak VGI strains were primarily associated with CNS disease in immunocompetent hosts, whereas outbreak strains (VGIIa and VGIIb) were more often involved with the lungs of patients with frequent underlying conditions. Evidence for increased virulence of outbreak strains was provided in mice models, in which some outbreak genotypes (notably VGIIa) were shown to be more virulent than others in vitro [43]. Interestingly, this did not simply appear to be linked to over-expression of individual pathogenicity factors in outbreak strains, but rather to an enhanced ability to proliferate in host macrophages [44]. This could perhaps help explain the high rates of lung cryptococcoma found in outbreak settings compared to reports from endemic cases.

## 5. *Cryptococcus gattii* Infections during Lymphoid Neoplasms

Haematological malignancy is a recognised predisposing factor for *Cryptococcus neoformans* infections, albeit to a lesser extent than solid-organ transplantation and receipt of systemic glucocorticosteroids [45]. Patients with lymphoid neoplasms appear to represent an emerging group of at-risk patients for cryptococccosis among those with haematological malignancy, which is comparable to what has been observed for invasive pulmonary aspergillosis in this population over the last decades [46,47,48]. Although haematological malignancy appears to be the most at-risk type of cancer for cryptococcosis [7], it only accounted for under 10% of 306 cases in HIV-negative patients [45]. There are only a few data on CG infections in the onco-haematological setting. In Australia, where CG is endemic, cases have been reported in patients with an increasingly wide range of immunocompromised states, with 8.5–27% having a non-HIV immunosuppressive condition [6,22]. However, only four cases of non-outbreak CG infections in patients with haematological malignancy have been specifically reported, to our knowledge [6]. With the British Columbia and U.S. Pacific Northwest outbreaks, new risk factors for CG infection were identified. Among 218 cases of CG infections in British Columbia from 1999–2007, 38 had a history of invasive cancer, including 13 patients with leukaemia or lymphoma [27]. Patients infected with outbreak strains in the Pacific Northwest were also more likely to have a pre-existing condition. Oral corticosteroid use was the most frequent condition associated with infections by these outbreak strains (accounting for 55% of cases), and receipt of corticosteroids within the preceding year was associated with risk of death. Among specific underlying conditions, only history of cancer was statistically more often associated with outbreak strains compared to non-outbreak strains [29].

We found no reported cases of CG in the literature specifically mentioning ibrutinib receipt in the treatment of lymphoid neoplasms. This is noteworthy, considering recent reports regarding the increased risk of invasive fungal infections in patients receiving ibrutinib, an inhibitor of the Bruton tyrosine kinase [49]. CLL is a malignancy of mature B cells, which exposes affected patients to an increased susceptibility to bacterial, mycobacterial, and fungal infections [50,51]. This is presumably related to both disease- and treatment-related factors. CLL, including in treatment-naïve patients, is characterised by quantitative and qualitative defects in B cells, T cells, NK cells, neutrophils, monocytes, and macrophages [52]. The overall incidence of invasive fungal infections remains low in CLL (estimated to range from 0.5 to 7.8% [53]), and susceptibility to cryptococcal disease in patients with B-cell malignancies seems to be particularly linked to advanced disease, pre-treatment, and longer duration of the underlying malignancy [45]. Patients receiving chemotherapy regimens containing fludarabine as well as the anti-CD52 monoclonal antibody alemtuzumab appear to be at particular risk of cryptococcal disease, presumably due to further quantitative and qualitative impairments in T cell responses [54,55]. More recently, there has been a particular focus on the epidemiological association between treatment regimens containing ibrutinib and the emergence of invasive fungal infections [49,56]. While the majority of these reported cases concerned invasive aspergillosis, *Cryptococcus neoformans* infections have also sporadically been reported in patients with ibrutinib-treated lymphoid neoplasms [56,57,58,59,60,61]. In 11 reported cases of CN cryptococcosis in patients with ibrutinib-treated CLL, 10/11 patients were male, and the median age was 67 years. Cryptococcosis occurred after a median of 15 (range 3–35) weeks after ibrutinib introduction, and 8/11 had prior or concurrent CLL treatments. The lungs were the most frequently affected organ (8/11), followed by the CNS (4/11) [49,58,59,60,62,63]. Susceptibility to infection in these ibrutinib-treated patients has been linked to altered B-cell receptor signaling and the inhibition of interleukin-2-inducible kinases [64], as well as impairments of innate immunity [65], most notably in neutrophil and monocyte functionality [66,67]. However, the fact that a significant number of reported cases of invasive fungal disease occurred in patients with relapsed/refractory diseases may point towards a combination of factors including disease and pretreatment, rather than being caused by ibrutinib alone [57].

Interestingly, it has been suggested that the CSF inflammatory response in cases of cryptococcal meningitis could be milder in patients with haematological malignancy [34]. Although this was not true in our reported patient, it was apparent in four previously reported cases of CN meningitis in patients receiving ibrutinib for whom clinical data were available (median CSF nucleated cell counts: 2/dL, range: 1–4/dL) [58,60]. This was also previously reported in cases of bacterial and fungal meningitis in patients with cancer [68], and is reminiscent of the non-inflammatory CSF profile sometimes found in cases of CN meningitis in profoundly CD4-depleted HIV/AIDS patients [69]. This particular aspect certainly deserves further investigation, with an emphasis on CG meningitis, which is characterised by significant CSF inflammation, and higher rates of hydrocephalus and neurological sequalae than CN [6].

## 6. Management of *Cryptococcus gattii* Infections in the Haematological Setting

Treatment guidelines for CG meningitis and meningoencephalitis are based on case series and expert opinions rather than controlled studies [5,70], but there nevertheless appear to be significant differences when compared to the management strategies for CN. Induction therapy regimens including a combination of amphotericin B (or L-AMB) and 5-FC for a duration of 6 weeks were associated with the highest success rates in cases of meningitis (although 2 weeks may be sufficient in cases of isolated pulmonary disease) [70]. Up to 18 months of maintenance treatment regimens with fluconazole are required in cases of CNS disease, which is longer than that recommended for CN meningitis [5]. Management of intracranial pressure is also fundamental, and often requires repeated lumbar punctures or the use of lumbar drains/CSF shunts [40]. Corticosteroid use can also be necessary in light of the high rates of IRIS-like manifestations, which can occur after 1 to 12 months [70]. One of the foreseeable challenges in the management of CG meningitis in the onco-haematological setting is the potential bone marrow suppression induced by 5-FC [71], which has been linked to the conversion of 5-FC to 5-fluorouracil by the intestinal microflora [72]. Indeed, in patients with an underlying haematological malignancy, particularly those having undergone radiation therapy or myelosuppressive chemotherapy, this adverse effect can lead to treatment cessation. Our case illustrates this, as induction therapy had to be prematurely discontinued due to neutropenia. Drug–drug interactions between first-line antifungal agents and targeted therapies can also be of concern. Azoles in particular interfere with the elimination of ibrutinib by inhibiting the CYP3A4 enzyme system, leading to an increased risk of adverse effects [73]. In all cases, the decision to continue or stop ibrutinib alongside azole-based antifungal therapy should be balanced against the risk of relapse of the underlying malignancy, especially when considering the prolonged duration of azole-based maintenance therapy. There are few data on the modalities of ibrutinib reintroduction in CLL when associated with azole-based therapies, but experts generally recommend marked dose reductions, usually at a quarter of the maximal prescribed dose (140 mg/d) [73,74]. Our case, however, illustrates that ibrutinib can safely be reintroduced alongside azole-based antifungals under strict monitoring of drug–drug interactions and signs of relapse of the underlying malignancy.

## 7. Conclusions

The clinical epidemiology, clinical presentation, and course of disease during infections caused by CG seem to involve complex interactions between environmental exposure to CG, infecting genotype, pathogen virulence factors, host susceptibility, and host immune responses. As the spectrum of underlying conditions predisposing individuals to CG infections and the geographical range of CG tend to expand, it is increasingly likely that sporadic cases of CG will be reported in patients with haematological malignancy. It is crucial for future guidelines to address the challenges involved in the management of CG in the onco-haematological setting, regarding both the duration of antifungal induction therapy as well as the management of drug–drug interactions between antifungals and targeted therapies.

## Data Availability

Data supporting the case report are available upon request to the corresponding author.
